# Organobeidellites for Removal of Anti-Inflammatory Drugs from Aqueous Solutions

**DOI:** 10.3390/nano11113102

**Published:** 2021-11-17

**Authors:** Eva Plevová, Silvie Vallová, Lenka Vaculíková, Marianna Hundáková, Roman Gabor, Kateřina Smutná, Radim Žebrák

**Affiliations:** 1 Institute of Geonics of the Czech Academy Sciences, Studentská 1768, 708 00 Ostrava, Czech Republic; lenka.vaculikova@ugn.cas.cz; 2 Department of Chemistry, VSB-Technical University of Ostrava, 17. listopadu 15, 700 30 Ostrava, Czech Republic; silvie.vallova@vsb.cz; 3 Institute of Environmental Technology, CEET, VSB-Technical University of Ostrava, 17. listopadu 15, 700 30 Ostrava, Czech Republic; katerina.smutna@vsb.cz; 4 Nanotechnology Centre, CEET, VSB-Technical University of Ostrava, 17. listopadu 15, 700 30 Ostrava, Czech Republic; marianna.hundakova@vsb.cz (M.H.); roman.gabor@vsb.cz (R.G.); 5 Dekonta Inc., Dřetovice 109, 273 42 Stehelčeves, Czech Republic; zebrak@dekonta.cz

**Keywords:** organobeidellite, sorption, ibuprofen, diclofenac, cetylpyridinium bromide, benzalkonium bromide, tetradecyltrimethylammonium bromide

## Abstract

Diclofenac (DC) and ibuprofen (IBU) are widely prescribed non-steroidal anti-inflammatory drugs, the consumption of which has rapidly increased in recent years. The biodegradability of pharmaceuticals is negligible and their removal efficiency by wastewater treatment is very low. Therefore, the beidelitte (BEI) as unique nanomaterial was modified by the following different surfactants: cetylpyridinium (CP), benzalkonium (BA) and tetradecyltrimethylammonium (TD) bromides. Organobeidellites were tested as potential nanosorbents for analgesics. The organobeidellites were characterized using X-ray powder diffraction (XRD), Infrared spectroscopy (IR), Thermogravimetry and differential thermal analysis (TG/DTA) and scanning microscopy (SEM). The equilibrium concentrations of analgesics in solution were determined using UV-VIS spectroscopy. The intercalation of surfactants into BEI structure was confirmed both using XRD analysis due to an increase in basal spacing from 1.53 to 2.01 nm for BEI_BA and IR by decreasing in the intensities of bands related to the adsorbed water. SEM proved successful in the uploading of surfactants by a rougher and eroded organobeidellite surface. TG/DTA evaluated the decrease in dehydration/dehydroxylation temperatures due to higher hydrophobicity. The Sorption experiments demonstrated a sufficient sorption ability for IBU (55–86%) and an excellent ability for DC (over 90%). The maximum adsorption capacity was found for BEI_BA-DC (49.02 mg·g^−1^). The adsorption according to surfactant type follows the order BEI_BA > BEI_TD > BEI_CP.

## 1. Introduction

Water resources responsible for the prosperity and development of various forms of life on earth are increasingly limited due to their contamination associated with the increasing generation of wastewater, whether industrial or domestic. The widespread use of various chemical compounds around the world leads to the serious pollution of water environment resources. A complex of hazardous chemicals such as heavy metals, polycyclic aromatic hydrocarbons, pharmaceuticals, pesticides, detergent compounds, organic dyes, adhesives, product of oil refineries or next products of chemistry and metal processing industries in contaminated soil, surface water and groundwater has brought long-lasting effects on human/animal health (toxic effects connected with immunotoxicity, reproductive impairment, teratogenicity or carcinogenicity) as well as negative effects on complex environmental balance [1,2,3]. One of the relatively new, alarming pollutants has recently been marked as pharmaceuticals such as analgesics, antibiotics, antidepressants or contraceptives. Due to the constant increase in their consumption, the pharmaceutical residues in the water are called new emerging pollutants [4,5,6]. Various physical, chemical and biological methods have been tested to remove drugs from wastewater, e.g., photocatalytic, ozonation, Fenton, biodegradable or sorption processes. However, conventional wastewater treatment plants rarely use tertiary or advanced treatment steps such as ultrafiltration, flocculation, ozonation or advanced oxidation processes with regard to their price. Adsorption is more common method due to its affordability, safety and possibility of sorbent regeneration [7,8].

Nanoparticles, called next generation nanoadsorbents, are able to remove emerging pollutants such as organic pharmaceutical residues even at low concentrations in micrograms per liter under various process conditions [9]. These nanocomposite materials based on clay minerals represent a significant development trend in new materials with predefined and required properties that predetermine their broad range of applications in sorption procedures [10,11]. Probably, the most used clay mineral for composite preparation is montmorillonite, but also a wider range of structurally similar and often neglected clay minerals of the smectitite type such as beidellite, nontronite, hectorite and saponite appear to be suitable for this preparation [12,13]. The basic structural unit of the smectite mineral is composed of units made up of two silica tetrahedral sheets with a central alumina octahedral sheet (2:1 structure). Isomorphic substitutions in the octahedral or the tetrahedral sheets render their layers anionic with compensating, usually hydrated, cations such as K^+^, Na^+^, Li^+^, Mg^2+^ and Ca^2+^ contained in the interlayer space. All the smectite minerals are identical in a basic structure but differ in chemical composition and the number of vacations in tetrahedral and/or octahedral sheets. Smectite minerals have a high cation exchange capacity, large specific surface areas (10–700 m^2^g^−1^) and exhibit a high swelling capability in the presence of H_2_O [14,15]. The clay mineral matrix serves as a basis for the modification/intercalation of various organic compounds, leading, subsequently, to the preparation of organo-clay composites. Generally, the modification process is based on replacing the interlayer cations with specific species to alter the surface and structural characteristics of the host clays. The principal modification process is related to the exchange of natural interlayer cations by organo-cations such as primary, secondary, tertiary and quaternary alkylammonium or alkylphosphonium cations. Such an ion exchange modifies the hydrophilic surfaces of the clay structure into organophilic surfaces, which enables the intercalation and sorption of the organic compounds [16,17,18]. Intercalated clay minerals—organically modified clay composites—thus, show a higher affinity for reactions with organic compounds or anions, in contrast to the original clay mineral. This property can be used to prepare adsorbents with a selective sorption capability for neutral non-polar organic compounds and anions, whether contaminants or natural substances compounds [19,20,21]. A search in the literature shows that clay minerals and their organically and/or inorganically modified forms do make it possible to successfully remove drugs from water and wastewater. For example, organoclays based on dodecylpyridinium chloride and hexadecylpyridinium chloride have been successfully applied as efficient adsorbents for diclofenac sodium from wastewater [22]. Next, authors solved the problems with the adsorption of psychoactive drugs onto sodium-exchanged montmorillonite under different pH conditions [23]. Diazepam was adsorbed through weak electrostatic interactions, whereas oxazepam appeared to be intercalated within Na-Mt layers, despite its neutral charge. On the contrary, codeine was protonated and its adsorption through cation exchange resulted in the highest adsorbed drug amount. Synthetized beidellites with various surfactants (tetraethylphosphonium, tetrabutylphosphonium, tetrahexylphosphonium and tetraethylphosphonium) were tested for their sorption capacity by loading sulfamethoxazole [24]. The adsorption of sulfamethoxazole was the most effective using beidellite modified by tetraoctylphosphonium. Additionally, other authors proved the possibility of the potential application of organoclays for removing pharmaceuticals from water such as analgesic, antibiotic or psychoactive drugs [25,26,27,28,29,30,31,32].

The objective of this study is the assessment of the ability of organo-beidellites to remove drugs from aqueous solutions due to the increasing wastewater pollution related to the high consumption of pharmaceuticals mentioned above. Beidellite, an often neglected and less common clay mineral, was modified with alkylammonium cations (BA, CP and TD) and prepared organobeidellites were checked as a potential sorbent for analgesic drugs (DC, IBU) from aqueous solutions. The organobeidellites were characterized using XRD, IR, TG/DTA, SEM along with UV-VIS. The results proved the possibility of their application for the removal of analgesics from an aqueous solution with a sufficient degree. Compared to organically modified montmorillonites, there is still a lack of information on the adsorption ability and capacity of organically modified beidellites; therefore, this study brings new and valuable information for the design of nanoadsorbents based on clay material.

## 2. Materials and Methods

### 2.1. Materials

**Figure 1:**
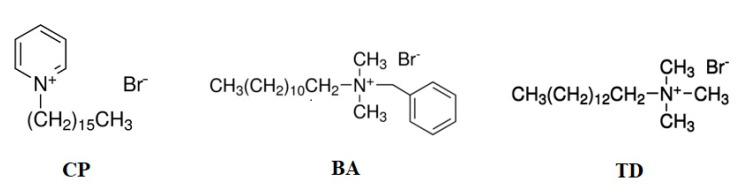
The structural formulas of used organic surfactants [34].

A beidellite sample (SBId-1) from an Idaho (USA) deposit was obtained from the Source Clays Repository of the Clay Minerals Society and signed as BEI. The cation exchange capacity (CEC) of this smectite clay mineral is 129 meq/100 g [33]. The material was purified by separating fine clay particles (≤5 μm) using sedimentation according to Stokes’ law. For the modification of beidelitte, cetylpyridinium bromide (CP) with a molecular weight of 358.31 g mol^−1^, benzalkonium bromide (BA) with a molecular weight of 384.44 g mol^−1^ and tetradecyltrimethylammonium bromide with a molecular weight of 336.39 g mol^−1^ were chosen. All three surfactants were supplied by Sigma Aldrich, USA. The structural formulas are presented in Figure 1.

**Figure 2:**
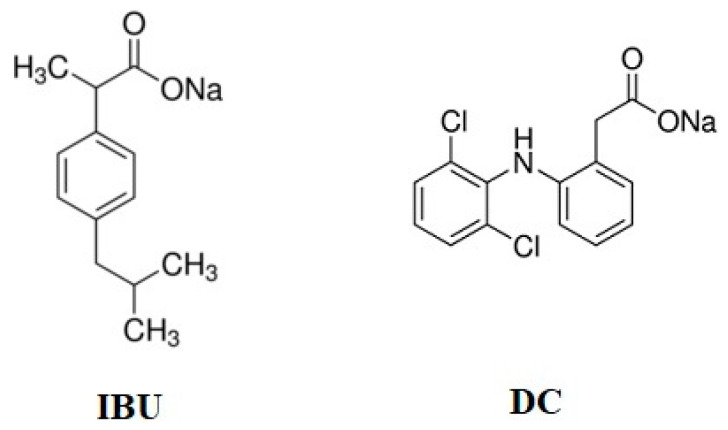
The structural formulas of used analgesics [34].

For adsorption procedures, two analgesic drugs, ibuprofen sodium salt (IBU), with a molecular weight of 228.26 g mol^−1^, and diclofenac sodium (DC), with a molecular weight of 318.13 g mol^−1^, were chosen; both were also supplied by Sigma Aldrich USA. The structural formulas are shown in Figure 2.

### 2.2. Methods and Equipment

The organically modified BEI samples were prepared according to following procedure. The BA, CP and TD salts were dissolved in water to obtain a 0.1 M solution. The suspension of BEI and water solution of BA, CP and TD were shaken thoroughly for 3.5 h at room temperature. Then, the suspension was centrifuged for 20 min and was re-suspended in an ethanol-water mixture. This suspension was centrifuged again and washed out with ethanol several times until it became free from excess salts and dried at room temperature. The samples were signed as BEI_BA, BEI_CP and BEI_TD.

Adsorption experiments were carried out in a batch mode, mass of sorbent was 100 mg, volume of IBU and DC solutions was 20 mL and initial concentrations varied from 40 to 300 mg L^−1^. The suspensions of sorbent with drug solutions were mixed on a rotary tumbler for 24 h. The samples were subjected to centrifugation and filtration. The amount of the drugs adsorbed onto organobeidellite at equilibrium was calculated using the following mass balance equation [35]:

(1) $$q_{e}=\frac{\left(c_{0}-c_{e}\right)V}{m}$$

where

*q_e_*—the sorption capacity/adsorbed amount of DC/IBU loaded to a unit amount of sorbent (mg g^−1^); 

*c_0_*—the initial concentration of DC/IBU in solution (mg L^−1^);

*c_e_*—the equilibrium concentration of DC/IBU in solution (mg L^−1^);

*V*—aqueous solution volume (L);

*m*—sorbent mass (g).

To determine the isotherm parameters, the following two linear equations of Langmuir and Freundlich were used [36]:

Langmuir

(2) $$\frac{c_{e}}{q_{e}}=\frac{1}{q_{max}\;K_{L}}+\frac{c_{e}}{q_{max}}$$

Freundlich

(3) $$ln\;q_{e}=ln\;K_{F}+\frac{1}{n}\;ln\;c_{e}$$

where

*c_e_*—the equilibrium concentration of DC/IBU in solution (mg L^−1^);

*q_e_*—the equilibrium adsorption capacity of DC/IBU loaded to a unit amount of sorbent (mg g^−1^);

*q_max_*—max. sorption capacity of DC/IBU loaded onto a unit amount of sorbent (mg g^−1^);

*K_L_*—Langmuir constant related to the free energy of adsorption; 

*K_F_*, *n*—Freundlich constants related to the adsorption capacity and adsorption strength.

The determination of major elements has been performed by means of a wavelength dispersive sequential X-ray fluorescence spectrometer ARL PERFORM’X 4200 W (Thermo Scientific, Waltham, MA, USA). The fusion bead method has been preferred for the preparation of pressed pellets in order to eliminate the heterogeneity due to grain size and a mineralogical effect and to reduce inter-element effects by dilution of the sample. Fused beads (40 mm) have been prepared by fusion of the samples with lithium tetraborate on a VULCAN 4M fusion machine (Thermo Scientific, Waltham, MA, USA) in Pt/Au crucibles.

X-ray powder diffraction (XRD) patterns were obtained using the Ultima IV diffractometer (RIGAKU, Tokyo, Japan) with CuKα radiation, a scintillation detector, a NiKβ filter and a Bragg-Brentano arrangement. Samples in standard holder were measured in an ambient atmosphere at operating conditions of 40 kV and 40 mA, a scanning rate of 2.0°/min and a scanning step of 0.02°·2θ.

The morphology of the particles of selected samples was studied using a Scanning Electron Microscope JEOL JSM-7610F Plus (JEOL, Tokyo, Japan) equipped with an autoemission gun as the electron source. The samples were scanned in a GENTLEBEAM mode (3 keV accelerating voltage) with the detection of the secondary electrons. Prior to the analysis, the sample was sputtered with an approx. 20-nanometer-thick Pt layer using a Quorum Q150V ES plus device (Quorum, Laughton, UK). The particles were examined at magnifications of 25,000×.

The infrared (IR) spectra of the original beidellite and its organically modified forms were recorded on a Nicolet 6700 FTIR spectrometer (Thermo Scientific, Waltham, MA, USA). Each sample (0.5 mg) was finely ground with 200 mg of dried KBr and compressed into a 13-millimeter diameter pellet using 7 tons of pressure under vacuum for 5 min. The spectra were collected from 4000 to 400 cm^−1^ in transmission mode by an accumulation of 64 scans with a resolution of 4 cm^−1^ and subsequently converted to a uniform weight of 1 mg.

Thermogravimetry and differential thermal curves (TG/DTA) were obtained using the thermal analyzer Setsys 24 Evolution (Setaram, Caluire-et-Cuire, France). Thermal curves were recorded under the following conditions: dynamic air atmosphere (20 cm^−3^ min^−1^); final temperature, 1200 °C; heating rate, 10 °C min^−1^; sample mass, 25 mg; thermocouple, Pt-Pt90/Rh10; inert reference material, Al_2_O_3_; no pressing in crucible (loose packed). 

The equilibrium concentrations of analgesics in solution were determined using UV-VIS with a Double-beam UV-VIS spectrophotometer UV-1800 (Shimadzu, Kyoto, Japan). The equilibrium concentrations of analgesics in aqueous solutions were measured at a wavelength of 222 nm for IBU and 275 nm for DC.

## 3. Results and Discussion

### 3.1. X-ray Fluorescence Analysis

The elemental composition (expressed in oxides) is shown in Table 1.

### 3.2. X-ray Powder Diffraction

**Figure 3:**
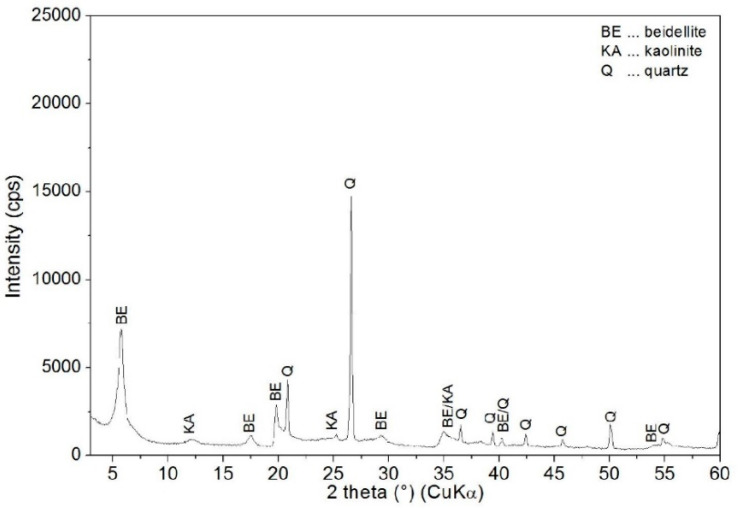
XRD pattern of unmodified BEI.

The XRD pattern of BEI (Figure 3) shows reflections of beidellite (PDF card no. 00-003-0013), quartz (PDF card no. 01-083-0539) and kaolinite (PDF card no. 01-072-2300) and also shows a basal (001) reflection at an angle 2θ 5.76° with an interlayer distance d = 1.53 nm.

**Figure 4:**
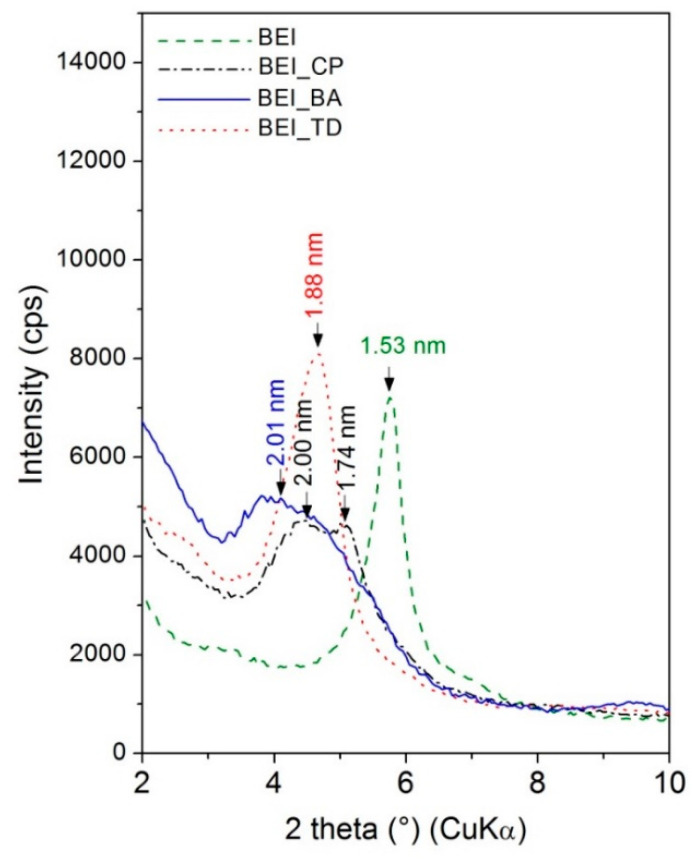
The comparison of XRD patterns of BEI, BEI_CP, BEI_BA and BEI_TD (from 2° to 10° 2θ).

After organic molecules intercalation, the BEI basal reflection was shifted to lower angle 2θ 4.40° with d = 2.01 nm for BEI_BA, to 2θ 4.68° with d = 1.88 nm for BEI_TD and to 2θ 4.42° and 5.08° corresponding to d-values of 2.00 and 1.74 nm for BEI_CP (Figure 4). These increases in the interlayer distance indicate a successful intercalation of all three organic molecules into the BEI interlayer space. Aa stable arrangement is supposed in a monolayer with a basal spacing of 1.30 nm, while a bilayer and a trilayer correspond to basal spacing of about 1.80 and 2.20 nm, respectively [37]. Therefore, the two reflections at sample BEI_CP may indicate different arrangements of CP molecules in the BEI interlayer. On the contrary, the sharper form of the BEI_TD peak reflects a more ordered intercalated structure in comparison with BEI_CP and BEI_BA. The higher intercalation indicated by the higher d_001_ value is caused by the system of π electrons in benzene and the pyridinium ring. These structure types are connected to the clay matrix both by electrostatic forces related to ion exchange and by other interactions between benzene rings and the clay surface or π-π interactions of the neighboring benzene nucleus [38,39].

### 3.3. SEM Analysis

**Figure 5:**
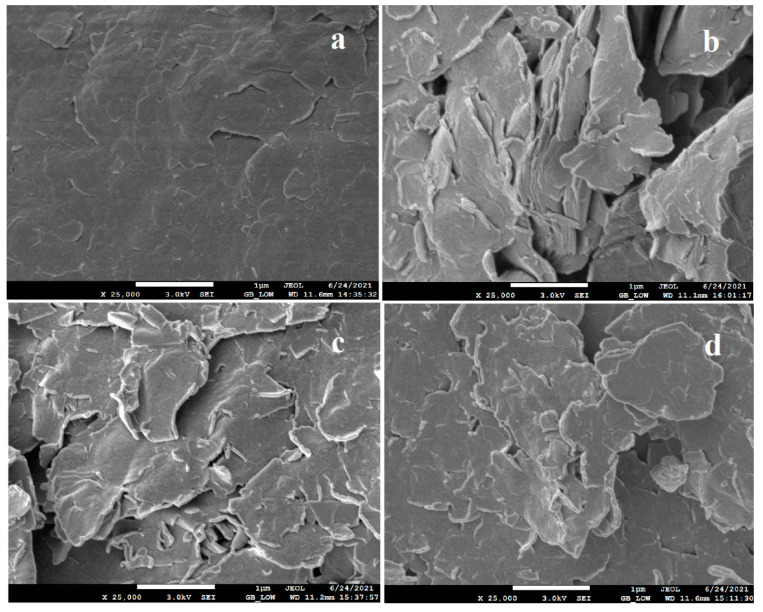
SEM images of: (**a**) BEI × 25,000; (**b**) BEI_BA × 25,000; (**c**) BEI_CP × 25,000; (**d**) BEI_TD × 25,000.

The surface morphologies of the unmodified and modified BEI were studied using SEM indicated in Figure 5. Photographs of the BEI samples were taken at 25,000× magnifications. The surface of the unmodified BEI is relatively smooth and plate-like (Figure 5a). Figure 5b–d show the surface of the modified BEI. It is evident that the surface became rougher, eroded and accompanied by small, wrinkled lamellae, which indicates the successful uploading of surfactants. The trend in the figures is similar to the SEM figures reported by other authors [40,41,42].

### 3.4. FT-IR Spectroscopy

**Figure 6:**
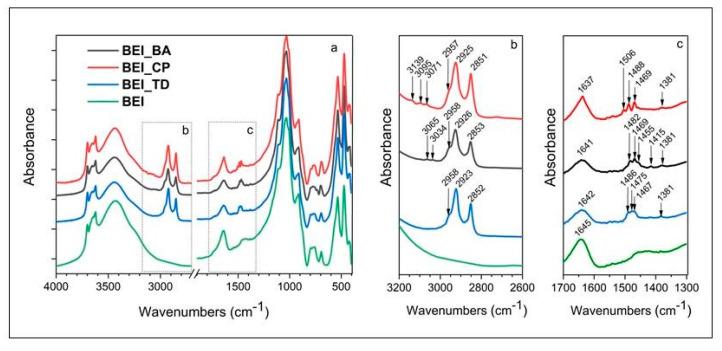
IR spectra of BEI_BA, BEI_CP, BEI_TD and BEI: (**a**) in the whole middle IR area, (**b**) from 3200–2600 cm^−1^ and (**c**) from 3200–2600 cm^−1.^

FT-IR spectroscopy was used to investigate the structural changes of modified beidellites. The IR spectra of BEI, BEI_BA, BEI_CP and BEI_TD are presented in Figure 6. Since beidellite belongs to dioctahedral smectites, its IR spectrum generally has a strong montmorillonite character [43]. Structural OH-stretching vibrations associated with octahedral Al-OH groups in beidellite structure are located between 4000 and 3500 cm^−1^. However, in the IR spectrum of the original beidelite (BEI), they overlap with the bands of kaolinite (3701, 3669, 3647 and 3622 cm^−1^), the presence of which was also revealed using XRD analysis (Figure 3). The related O-H bending vibrations occur at 936 cm^−1^ (Al-OH) and 913 cm^−1^ (Al-OH). Similar spectral bands were reported [44] around 935–965 and 913–920 cm^−1^ for the Al-OH in-plane modes in all the dioctahedral species. The adsorbed water is evidenced by an O-H stretching vibration at 3425 cm^−1^ and the associated H-O-H bending vibration located at 1645 cm^−1^. The strong absorption band at 1031 cm^−1^ belongs to a Si-O-Si stretching vibration. Toward the lower wavenumbers, lattice deformation vibrations are manifested, namely, the bands at 535 cm^−1^ (Al-O-Si), 473 cm^−1^ (Si-O-Si) and 423 cm^−1^ (Al-O-Al), which were mentioned in the works [24,45]. In addition to the admixture of kaolinite, quartz also occurs in the beidelite sample (798, 779 cm^−1^). The existence of organic surfactants is well readable in the IR spectra through their characteristic functional groups. As can be seen from Figure 6a,b, the loaded organic molecules were most pronounced in the spectral range 3200–2600 and 1650–1200 cm^−1^. All the prepared organobeidellites (BEI_BA; BEI_CP and BEI_TD) have a typical doublet of bands between 3200 and 2600 cm^−^^1^, which is caused by the C-H stretching vibrations. Specifically, it is an absorption band lying around 2925 cm^−1^ and a band at 2852 cm^−1^, which belong to the asymmetric and symmetric stretching vibrations of the methylen (–CH_2_) groups from the alkyl chain of surfactant molecules. A weak shoulder toward the higher wavenumbers at 2958 cm^−1^ corresponds to the asymmetric C-H stretching of the terminal methyl (–CH_3_) groups. The related deformation of these groups can be found at 1469 (–CH_3_ asymmetric), 1455 (–CH_2_ scissors) and 1381 cm^−1^ (–CH_3_ umbrella). The presence of aromatic C-H groups (for sample BEI_BA and BEI_CP) is indicated by the stretching vibrations of C-H aromatics whose bands lie above 3000 cm^−1^. The C-C skeletal vibrations of the aromatic ring are manifested by a slight at 1501 cm^−1^. However, this band is distinguishable only in sample BEI_CP. The above spectral bands indicate that the surfactant molecules have been successfully adsorbed into an interlayer space of beidellite. The comparison of the IR spectra of organobeidellites with the unmodified clay mineral shows that the clay matrix remains unchanged. The only exceptions are bands of physically adsorbed water, the absorption of the organic molecule caused a decrease in their intensities and a smaller shift in their positions. Figure 6b demonstrates this fact on the deformation vibration of the O-H groups of water. The basic position of the bend 1645 cm^−1^ (BEI) gradually shifts toward the lower wavenumbers 1642 (BEI_TD), 1641 (BEI_BA) up to 1637 (BEI_CP). The weaker absorbances at 3425 and 1645 cm^−1^ in the IR spectra of all the organically modified forms correlate well with the results of the TG/DTA analysis, where a large amount of absorbed water has been detected in the interlayer space, while after intercalation the amount of water decreased.

### 3.5. TG/DTA

**Figure 7:**
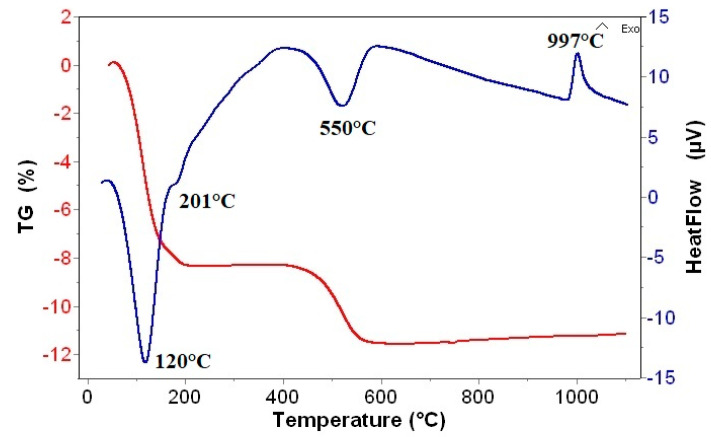
TG/DTA curve of BEI.

**Figure 8:**
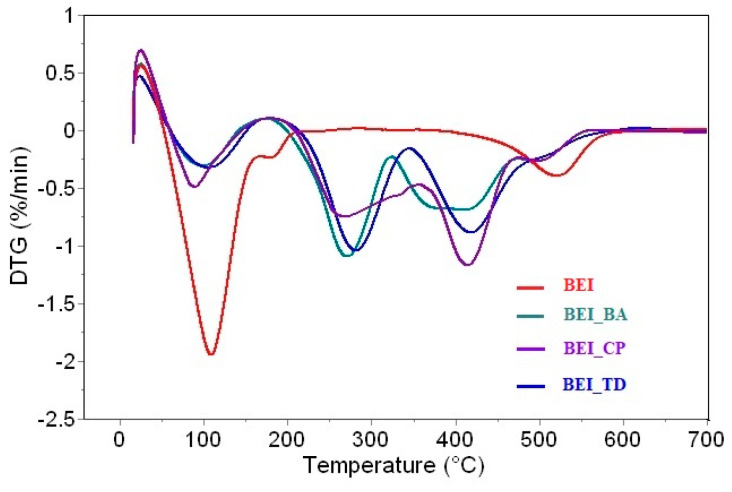
DTG curves of BEI, BEI_CP, BEI_BA and BEI_TD.

The differential thermal curves show the endothermic and exothermic processes during heating in both the unmodified and modified BEI samples. The measured DTA curves of BEI showed a double endothermic peak at 120 and 201 °C connected to of the release of adsorbed water and water between the layers (Figure 7). Then, the constitution water at 550 °C was released (middle endothermic peak). The crystal lattice was broken, and final dehydrated substance was recrystallized and transformed, accompanied by a small exothermic peak. The transformation’s products can be variable mineral mixtures of mullite, spinel, cristoballite or cordierite, which are typical products for minerals from the smectite group. In case of modified beidellites, in addition to the reactions associated with the decomposition of clay matrix, the decomposition of the added surfactant is also manifested on thermal curves. Figure 8 shows the DTG curves of unmodified and modified beidellite samples. For BEI, the first two DTG peaks are associated to mass loss due to the desorption of water (at 110 and 180 °C), the third peak at 521 °C is related to the dehydroxylation process. For BEI_BA, the first peak at 97 °C belongs to the release of adsorbed water, the next three peaks belong to mass loss connected with the degradation of BA (at 269, 374 and 410 °C) and the last peak at 499 °C is related to the dehydroxylation of the clay matrix. For BEI_CP, the first peak at 95 °C is related to water desorption, the next three peaks also belong to mass loss due to the degradation of CP (at 268, 331 and 414 °C) and the dehydroxylation of clay matrix is connected to the peak at 502 °C. For BEI_TD, the water desorption comes with the peak at 105 °C, the mass loss connected with the degradation of TD is the only double step process (at 282 and 418 °C) and the mass loss due to releasing constitution water is connected to the peak at 496 °C. The obtained results showed that the intercalation process caused a decrease in both the dehydration and dehydroxylation temperatures due to an increase in the hydrophobicity after organic modification, which also corresponds with the results of other authors [46,47,48]. This behavior is caused by an increase in the hydrophobicity of the clay mineral after modification by surfactant. This process reduces the water content of the modified clay, which also reduces the dehydration temperature. This is probably due to the penetration of methyl groups of surfactants into the siloxane layers, which make it possible to remove OH groups in the form of water vapor [49,50].

### 3.6. Sorption Study

**Figure 9:**
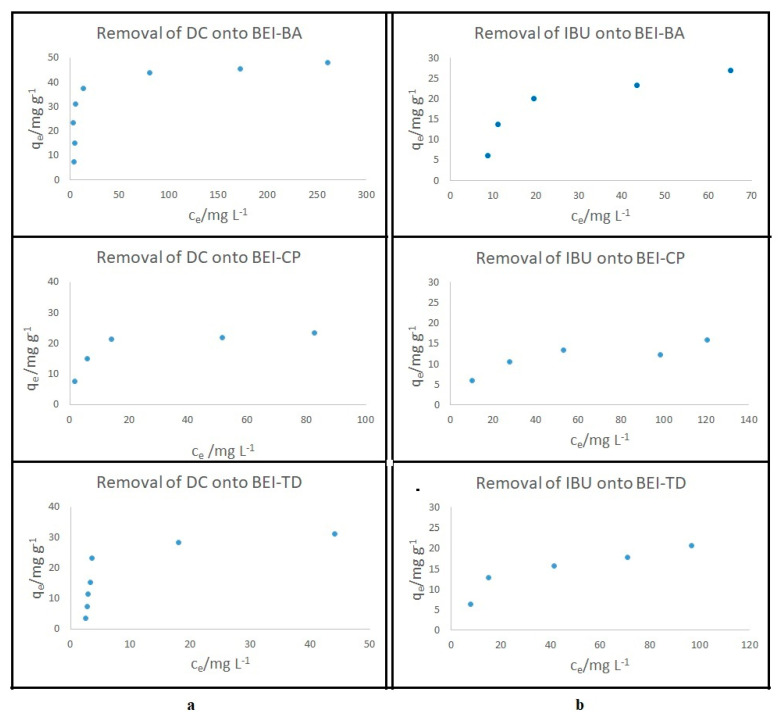
Sorption isotherms (**a**) for DC and (**b**) for IBU.

The sorption experiments for the removal analgesics DC and IBU from aqueous solutions were carried out on modified beidellites. The hydrophobic organic molecules, such as DC and IBU, are sparingly soluble in water; therefore, it is assumed that they will be sorbed at the hydrophobic end group of the surfactants. The equilibrium concentrations of drugs in solution were determined using UV-VIS at λ = 275 nm for DC and λ = 222 nm for IBU. The amount of DC and IBU adsorbed onto beidellites was determined from the difference between the initial concentration of analgesics in the solution and the equilibration concentration using Equation (1). The obtained isotherms of analgesics are illustrated in Figure 9. These isotherms were obtained by plotting the amounts of the drugs adsorbed per weight unit of each sorbent against the equilibrium concentrations of the analgesics in the solution. The values of the sorption capacity ranged from 25 to 50 mg g^−^^1^ for DC and from 15 to 30 mg·g^−^^1^ for IBU. The highest adsorbed amount (47.8 mg·g^−1^) was achieved for DC uptake onto BEI_BA, when the equilibrium was established at a higher initial concentration compared to the other sorption tests. In contrast, the lowest values of adsorption were calculated for BEI_CP for both drugs. The efficiency removal onto BEI_CP for IBU was 55% and for DC was 91%. This fact is probably related to the different arrangement of CP molecules in the BEI interlayer space, which was proved by two reflections obtained using XRD (Figure 4). The removal of DC was more efficient (over 90%) than IBU (55–86%) for all the modified sorbents (BEI_CP, BEI_BA and BEI_TD). The higher removal of DC is a consequence of a combination of electrostatic (i.e., Coulomb) interactions between the quaternary ammonium of the modified clay and the ionized carboxylic acid of the drug DC containing two benzene rings, which leads to both higher polarizability and the greater dispersive interactions in contrast to IBU [51]. The experimental equilibrium data were evaluated by using the Langmuir and Freundlich isotherms, the most frequently employed models to quantify the adsorption capacity of the adsorbents for the removal of drugs. The adsorption equilibrium is described as a function of the adsorbed amount on the concentration at a constant temperature. The Langmuir adsorption isotherm, originally developed to describe the adsorption of the gas-solid phase on activated carbon, is now generally used to quantify various type of sorbents. The Langmuir model is based on the following theoretically assumption: only one layer of molecules is formed, the probability of adsorption is the same at all sites on the surface and the adsorbed molecules do not interfere with each other. While Freundlich’s model is the oldest known relation describing the non-ideal and reversible adsorption, this empirical model can be applied to multilayer adsorption with uneven distribution of adsorption heat on the heterogeneous surface. The model is based on the adsorption on heterogeneous surfaces where the interaction between the adsorbed molecules is not limited to monolayer formation [52,53]. The constants of the Langmuir and Freundlich isotherms (Table 2) were obtained from two regression curves based on the linear equations of Langmuir and Freundlich (Equations (2) and (3)).

The Langmuir model gave a better fit than the Freundlich model on the basis of the correlation coefficient values R^2^ (0.94–0.99 for Langmuir and 0.82–0.92 for Freundlich). The maximum adsorption capacity in the monolayer obtained from the Langmuir isotherm was the highest for BEI_BA-DC (49.02 mg g^−1^). The Langmuir isotherm was found to describe the equilibrium sorption data well over the entire concentration range.

The results of the sorption processes published in the literature are generally performed under various experimental conditions such as different initial concentrations, mass of sorbent or volume of aqueous solutions; therefore, comparing the materials used for sorption is complicated and can be misleading. The comparison of parameter such as the sorption capacity *q_e_* may differ according to the initial experimental conditions. The available literature shows that cheap biomaterials such as sorbents lead to *q_e_* values of the order of 10^−2^–102 mg g^−1^. However, in comparison with commercially available sorbents, clay minerals belong to low cost and environmentally friendly materials [54]. An important parameter of the used sorbents is the possibility of their regeneration. The regeneration processes can be divided into thermal, extraction, chemical and others. The regeneration process should be easy controlled, low energy efficient and with a minimal harmful impact on the environment [55]. Sorbent regeneration processes are predominantly financially disadvantageous for “low-cost” adsorbents such as clay minerals. In addition, the thermal treatment of these type of materials would lead to their structure and texture destruction.

## 4. Conclusions

The modification of the beidellite sample with three different surfactants (BA, CP and TD) was performed. XRD confirmed the intercalation of all three surfactants into the BEI structure by the increase in basal spacing. IR spectroscopy proved the absorption of the surfactant molecules by the decrease in intensities and the positions of bands related to physically adsorbed water. TG/DTA confirmed the presence of surfactants due to an increase in the hydrophobicity accompanied by a decrease in the dehydration temperature. The removal of DC was more efficient (up to 90%) than IBU (55–86%) for all the organobeidellites. The maximum adsorption value of 49.02 mg g^−1^ obtained from the Langmuir isotherm was found for BEI_BA-DC. The results suggest that the modification of beidellites can lead to the preparation of organobeidellites as potential inexpensive nanosorbents for non-steroidal anti-inflammatory drugs from aqueous solutions. The efficiency of sorption is sufficient with respect to the relatively low concentrations of pharmaceuticals in wastewaters.

## Figures and Tables

**Table 1:** Chemical composition of BEI sample.

Oxides	BEI (wt%)
Na_2_O	1.36
MgO	0.49
Al_2_O_3_	21.41
SiO_2_	60.10
P_2_O_5_	0.025
K_2_O	0.95
CaO	0.69
TiO_2_	0.75
MnO	0.008
Fe_2_O_3_	1.50

**Table 2:** Isotherm parameters of IBU and DC onto modified beidellites.

Langmuir				Freundlich		
	R^2^	qmax/mg g^−1^	K_L_/L mg^−1^	R^2^	K_F_/mg^−1−1/n^ L^1/n^ g^−1^	n
BEI_CP—DC	0.9982	24.04	0.29861	0.8406	8.18497	3.78
BEI_BA—DC	0.9962	49.02	0.11538	0.8752	1.27647	1.43
BEI_TD—DC	0.9799	36.63	0.14138	0.8235	7.03572	2.29
BEI_CP—IBU	0.9479	16.67	0.05658	0.8785	2.94527	2.87
BEI_BA—IBU	0.9925	32.26	0.07188	0.9288	6.41154	2.87
BEI_TD—IBU	0.9838	23.87	0.05249	0.8741	3.31679	2.45

## Data Availability

Not applicable.
